# Trends of Late Presentation to Care in Patients with Chronic Hepatitis C during a 10-Year Period in Croatia

**DOI:** 10.3390/idr12030016

**Published:** 2020-11-11

**Authors:** Neven Papic, Leona Radmanic, Davorka Dusek, Ivan Kurelac, Snjezana Zidovec Lepej, Adriana Vince

**Affiliations:** 1 Department of Viral Hepatitis, University Hospital for Infectious Diseases, 10 000 Zagreb, Croatia; drnevenpapic@gmail.com (N.P.); davorka.dusek@gmail.com (D.D.); kurelacivan@gmail.com (I.K.); avince@bfm.hr (A.V.); 2 School of Medicine, University of Zagreb, 10 000 Zagreb, Croatia; 3 Department of Immunological and Molecular Diagnostics, University Hospital for Infectious Diseases, Mirogojska 8, 10000 Zagreb, Croatia; leona.radmanic@gmail.com

**Keywords:** chronic hepatitis C, HCV, liver cirrhosis, late presenters, end stage liver disease

## Abstract

Late presentation to care is the major obstacle to receiving treatment for chronic hepatitis C (CHC). Our aim was to analyze the prevalence and trends of late presenters (LP) at first consultations in Croatia during a 10-year period. This retrospective cross-sectional study included all adult CHC patients (*n* = 854) entering specialist medical care at the University Hospital for Infectious Diseases Zagreb between 2009 and 2018. LP was defined as liver stiffness measurement ≥ 9.5 kPa or biopsy METAVIR F ≥ 3. During the study period, mean patients’ age increased from 37 to 52 years while HCV genotype distribution changed leading to the replacement of genotype 1b with 1a (g1b 32% to 21%; g1a 19% to 38%). A total of 320 (37.4%) were LP; they were older (47.5, IQR 40.5–57.6), and more commonly infected with g1b (34.1%) and g3 (42.5%). The prevalence of LP significantly increased from 31.9% in 2009 to 46.5% in 2018. Late presentation for care of CHC is increasing in Croatia suggesting a gap of diagnosing strategies in patients over 50 years.

## 1. Introduction

Chronic hepatitis C (CHC) continues to be a major public health problem and leading cause of liver cirrhosis and hepatocellular carcinoma [1,2]. World health organization (WHO) has set a goal to reduce new viral hepatitis infections by 90% and to reduce deaths due to viral hepatitis by 65% by 2030, which now seems achievable by the treatment with highly effective direct acting antiviral agents (DAA) [3]. However, linkage to care is the main obstacle to receiving treatment and follow-up for liver disease and, consequently, a significant proportion of chronically infected patients enter care only after developing significant fibrosis or cirrhosis [4,5]. In the absence of widespread screening programs, diagnosing CHC may therefore be based on signs of late stage liver disease, which represents the main barrier in achieving WHO goals. In 2015 EASL endorsed consensus definitions on late presentation to care of viral hepatitis in order to determine the size of the population at risk and to identify vulnerable groups and risk factors for late presentation with the goal to improve the prevention and control responses to the viral hepatitis epidemic [6].

In Europe, reported data indicate that HCV predominantly affects men aged 25–44 years, which is consistent with the demographic profile of injecting drug use, the main route of transmission [7]. In Croatia, prevalence of CHC in the general population is 0.9% [1,8,9]. However, the prevalence of up to 65% has been reported in risk groups, mainly intravenous drug users (IDU) [8,10].

Incidence of newly discovered anti-HCV positive persons in Croatia during the 1990s increased steadily with the 400 newly diagnosed patients annually form 2000 to 2007 [11]. After 2008 there is a gradual decline and today there are up to 200 of newly discovered anti-HCV positive individuals per year [11]. In a recent retrospective cohort study, increased proportions of patients with advanced stages of fibrosis and cirrhosis among elderly patients (≥60 years of age) compared to the total cohort as well as increased frequency of high fibrosis index based on four factors (FIB-4) score [12]. Literature data on late presenters to care in Europe are available only for several EU countries. However, no information for Croatia or countries in the region has been reported so far.

The aim of this study was to investigate the prevalence and trends of late presenters for care (LP) at first consultations in Croatia during a 10-year period.

## 2. Methods

Croatia is a country with universal access to healthcare. DAA-based treatment of CHC is available free-of-charge, pending the approval by the National Insurance Fund. Eligibility for the treatment is based on the Recommendations for the Treatment of Hepatitis C by the Croatian Reference Center for Diagnostics and Treatment of Viral Hepatitis, Ministry of Health, R. of Croatia. Currently, organised national programmes for hepatitis C screening are not available. Referral-based HCV testing (serological and molecular) is available in clinical hospitals and transfusion centers. Voluntary, anonimous, free-of-charge testing (by using rapid HCV assays) is available in VTC centers operated by non-governmental organisations.

We conducted a retrospective cross-sectional study that included all adult CHC patients at their first contact with medical care at the Department of Viral Hepatitis University Hospital for Infectious Diseases Zagreb between January 2009 and December 2018. Excluded were patients with HIV and/or HBV co-infection. Patients were stratified by fibrosis stage into two groups: Late presenters (LP) and non-late presenters (non-LP). Late presenters for care were defined as a reliable LSM ≥ 9.5 kPa, APRI > 1.5, FIB-4 > 3.25 or biopsy METAVIR ≥ F3, according to EASL definition [6]. Presentation with late stage liver disease (LSDL) was defined as the presence of at least one symptom of decompensated cirrhosis (jaundice, hepatic encephalopathy, clinically detectable ascites, variceal bleeding) and/or hepatocellular carcinoma in patients with no previous antiviral treatment [6].

Records of all patients were extracted and used for collection of clinical and laboratory data. HCV RNA quantification was performed by COBAS Ampliprep/COBAS TaqMan HCV test (Roche Diagnostics, Diagnostic Systems, Pleasanton, CA, USA). HCV genotyping was performed by VERSANT HCV Genotyping assay (LIPA, Bayer Diagnostics, Puteaux, CEDEX, France). From 2009 to 2014 liver biopsies were performed and since 2015 fibroelastography has become a standard method. Liver biopsy was performed in total of 417 patients and the Ishak scoring system was used as an indicator of histological activity [13]. Liver stiffness measurement was performed with the FibroScan device (Echosens, France), and fibrosis was classified as: F1 < 7.0 kPa, F2 7.0–9.5 kPa, F3 > 9.5 kPa and F4 > 11.5 kPa.

Late presentation over time, genotype distribution, risk factors for transmission, age and sex distribution were analyzed for each year. Risk factors for late presentation were evaluated. Alcohol consumption was not assessed due to the incomplete data. IDU as a risk factor was defined as ever having a self-reported episode of injecting. The majority of IDU patients described themselves as former drug users.

Data were presented and evaluated descriptively. χ^2^ with Yates correction, Fisher’s exact test and Mann Whitney U test were used to compare the groups, as appropriate. All tests were two-tailed; a *p* < 0.05 was considered statistically significant. This study was conducted according to the ethical guidelines of the Declaration of Helsinki and was approved by the UHID Ethics Committee.

## 3. Results

We identified 854 newly diagnosed patients with CHC who were eligible for the study. The majority of patients were males (537, 62.8%) with median age of 39 years (IQR 34–54). During the period studied, patients’ age increased from 37 (IQR 30–52) in 2009 to 52 (IQR 44–54) years in 2018. There was no change in sex distribution during time.

The data on risk factor was available for 739 (86.5%) patients; in 216 (29.2%) patients the risk factor was reported as unknown. The main known risk factor was intravenous drug use (370, 50.1%), followed by blood transfusion and/or surgery (116, 15.7%), tattoo or piercing (18, 2.4%) and sex (19, 2.6%). There were no significant changes in reported risk factors over time.

**Figure 1:**
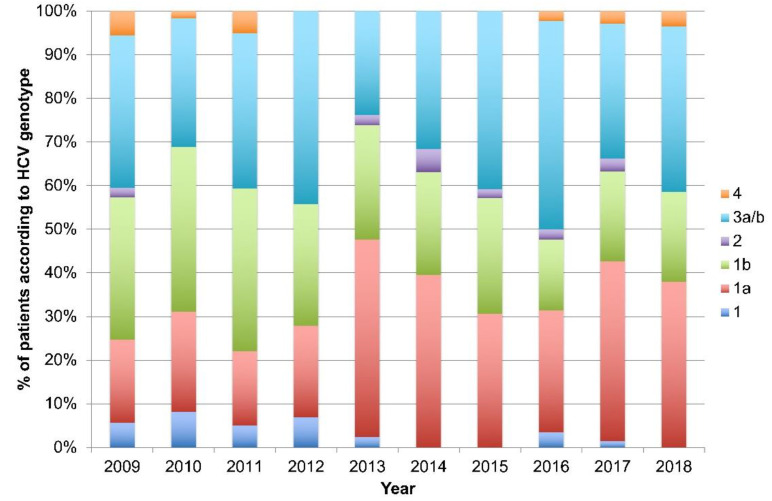
Genotype distribution in newly diagnosed patients with CHC.

However, there was a change in HCV genotype distribution; replacement of genotype 1b with 1a from 2009 to 2018 (g1b 32% to 21%; g1a 19% in to 38%). The incidence of genotype 3a/b from remained stable over time, compromising one third of the population, as presented in Figure 1.

Shown are proportions of HCV genotypes in newly diagnosed patients with CHC between 2009 and 2018 in Croatia.

A total of 320 (37.4%) patients were late presenters. Late presenters were more commonly men (204, 69.6%), older (47.5, IQR 40.5–57.6), and more commonly infected with HCV genotypes 1b (34.1%) and 3 (42.5%), as shown in Table 1. Similarly as in non-LP, there was change in genotype distribution over time; increase in subtype 1a and decrease in subtype 1b. Among people above the age of 50, 51.4% were LP. There were no differences in risk factors between LP and non-LP group during the studied period.

**Figure 2:**
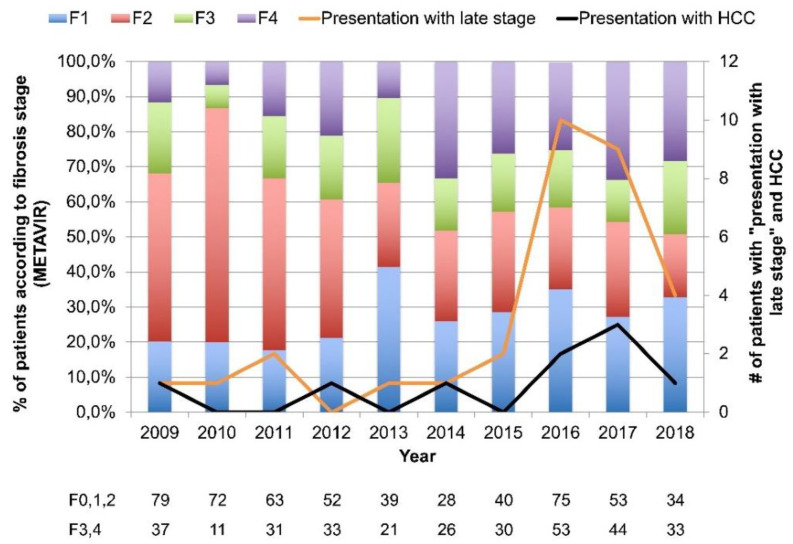
Trends in late presentation to care and presentation with the late stage of liver disease.

Importantly, the prevalence of LP significantly increased during the studied period from 31.9% in 2009 to 46.5% in 2018, as presented in Figure 2. As stated above, this correlates with increasing age, duration of infection and increase in the proportion of genotype 1a.

Presented are proportions of patients according to fibrosis stage (METAVIR) and number of patients presenting with late stage liver disease and HCC between 2009 and 2018 in Croatia.

Furthermore, there were 31 (3.6%) patients presenting with LSLD (31 of 320 of the LP patients, 9.7%). Majority of them were males (24, 77.4%), and their median age was 54.4 years (IQR, 45.0–60.4, none of the LSLD patients were under 40 years of age. The median liver stiffness measurement was 51.4kPa (IQR 35.3–69.0), 15 (48.4%) had genotype 3, 10 (32.2%) subtype 1b and 6 (19.3%) subtype 1a HCV infection. Of them, 9 patients had HCC diagnosed during the first 6 months (6 males, median age of 59.0, IQR 55.6–65.2 years). However, 23 of 31 (74.2%) LSLD patients were diagnosed after 2016, suggesting an increasing trend.

Overall, 649 patients received treatment (75.92%), 44 (5.1%) were neglected to treatment, 51 (5.9%) are waiting for treatment and 110 were lost to follow up (12.8%). Reasons for lack of treatment included active alcohol/drug abuse (15, 34%), contraindications for PegIFNα + RBV (10, 22.7%), limited life expectancy (6, 13.6%) and age limitation for treatment according to National Insurance Fund (13, 29.5%).

Until 2015, 525 patients were treated with pegylated interferon alpha (PegIFNα) and ribavirin (RBV), with sustained viral response rates (SVR) of 57.5%. DAAs were administered as first therapy in total of 124 patients (SVR rate of 98%) from 2015 to 2017 with prioritization for F3 and F4 fibrosis stage and from 2018 without prioritization.

## 4. Discussion

Here we present the results of a 10-year cross-sectional study of HCV monoinfected patients that have presented for the first time to specialist care at Croatian Referral Center for Viral Hepatitis, the largest national viral hepatitis treatment facility that cares for approximately 50% of the patients in Croatia.

Diagnosing CHC at earlier fibrosis stage has been shown to be crucial for achieving the WHO strategy goals [4]. Late presenters to care have a higher risk of developing HCC and achieving SVR decreases, but do not eliminate risk of HCC in those patients [14,15]. Therefore, CHC treatment initiation is prioritized to patients with advanced fibrosis [16].

Currently, treatment of CHC with direct acting antivirals (DAAs) is available in the majority of the EU countries. Still, the exact data on disease burden are missing from most of the countries. Therefore, national action plans rely on incidence reporting as well on the few seroprevalence studies among general population and risk groups. There is very few data on the prevalence of fibrosis in patients who are newly diagnosed with CHC and the effect of the DAAs on reduction of late presentation to care is still unknown.

At our tertiary care center we have performed fibrosis evaluation in all patients that presented for first specialist consultation, as it was mandatory to start the process of therapy approvement by National Insurance Fund. Therefore, we were able to assess the proportion of patients presenting with advanced liver disease for each year and monitor the trends over time. Overall 37.5% of patients were late presenters, and the prevalence of LP significantly increased during the studied period from 31.9% in 2009 to 46.5% in 2018. This coincided with the increase in age and changes in genotype distribution.

In contrast, in the German cohort that included 653 HCV monoinfected patients, the prevalence of LP was already decreasing to 26.4% in the year 2017 [17]. Genotype 3 remained an independent risk factor for LP and this might be due to the late introduction of DAA treatment for genotype 3 [17]. This is probably also linked with a decrease in the proportion of patients with CHC receiving liver transplantation after the introduction of DAAs in Germany [18].

The data from a Danish single-center study that included 570 patients from 2007 to 2016 found 32.5% of LP, but with no statistically significant change over time [19]. The high proportion of LP was also recently reported from Australia, with 16.5% of patients with advanced fibrosis (≥12.5 kPa) and 24.6% with liver stiffness measurement between 8.0 and 12.5 kPa [20]. In an Indian cohort study that included 777 patients that entered care between 2008 and 2014, 56% of patients already had cirrhosis at presentation [21]. However, trends over time have not been monitored and with generic drugs being available widely in India for several years there might be a change in the proportion of LP.

The importance of early diagnosing of CHC is emphasized in Chronic Hepatitis Cohort Study (CHeCS), a large American observational study; 17% of patients had been diagnosed with advanced liver disease concurrent with their initial HCV diagnosis, despite the fact that they have been on an average of 6 years in the health system before their HCV diagnosis [22]. These patients had high rates of hospitalization (59%) and mortality (33%), highlighting the severe consequences of missed opportunities for earlier diagnosis [22].

In addition to high prevalence of LP in our cohort, we have found significant change in genotype distribution; while genotype 3 remains most common, the proportion of genotype 1a increases over the last 5 years both in LP as in non-LP. This might be relevant since there are important genotype-specific differences in progression to cirrhosis and in response to different DAA treatment regiments [23,24].

Importantly, the number of patients with history of former and current IDU is still high, as in studies mentioned above, and within we find patients with genotypes 1a and 3 that were infected at different times, have different duration of infection and fibrosis stage. Regarding large number of patients with unknown risk factor there are probably many patients with history of shorter IDU practice in the past that do not want convey those details to the doctors. The significant number of patients (12.8%) “drop off” from our cohort and they might have been at risk for developing complications of CHC thus representing an another area for improvement of cascade of care. So the large efforts in national action plan should be at the level of primary care physician who should interview their patients regularly for the possibility of HCV infection.

In conclusion, we have shown that there is high burden of advanced liver disease in Croatia that was previously underestimated, with increasing trends of late presenters. Although the DAA treatment has been in place since 2015, still there is no impact on number of LP. These data suggest that to achieve WHO goals, there is an obvious need to increase the screening and linkage to care.

## Figures and Tables

**Table 1:** Baseline patients’ characteristic.

	Late Presenters (*n* = 320)	F0–F2 (*n* = 534)	*p*-Values ##
Age, median, IQR	47.5 (40.5–57.6)	38.8 (32.7–521.1)	0.0001
<30 years	18 (5.6%)	77 (14.4%)	0.0001
30–40 yr	56 (17.5%)	206 (38.6%)	0.0001
40–50 yr	94 (29.4%)	108 (20.3%)	0.0027
50–60 yr	91 (28.4%)	75 (14.0%)	0.0001
60+ yr	61 (19.1%)	68 (12.7%)	0.0137
Male sex	204 (69.6%)	301 (56.4%)	0.0371
Years since diagnosis *	24.4 (14.5–28.6)	12.2 (7.8–21.3)	0.0001
Risk factor #			
Intravenous drug use	149 (48.5%)	221 (51.2%)	0.5021
Blood transfusion/surgery	50 (16.3%)	66 (15.3%)	0.7584
Tattoo/piercing	6 (2.0%)	12 (2.8%)	0.6297
Sex	6 (2.0%)	13 (3.0%)	0.4816
Unknown	96 (31.3%)	120 (27.8%)	0.3250
Genotype			
1	4 (1.3%)	33 (6.2%)	0.0004
1a	67 (20.9%)	169 (31.6%)	0.0007
1b	109 (34.1%)	127 (23.8%)	0.0015
2	2 (0.6%)	11 (2.1%)	0.1473
3a/3b	136 (42.5%)	177 (33.1%)	0.0067
4	2 (0.6%)	17 (3.2%)	0.0149

* Data were available for 594 patients; # data were available for 739 patients; ## Fisher’s exact or Mann Whitney U test, as appropriate.
